# Cases Reported to the Chicago Medical Society

**Published:** 1866-03

**Authors:** Charles G. Smith


*Cases Reported to the Chicago Medical Society.* By Dr. CHARLES G. SMITH.

I. HÆMATURIA IN A NEW-BORN CHILD.

Mrs. N—— was delivered, after a somewhat protracted and severe labor, on the 20th ult., of a large and fine looking male child. The head came down well into the pelvis, but after three or four hours of very severe pains, did not advance, and I used the forceps, putting a speedy end to the labor, and without any apparent injury to the child. On the evening of the 23d, however, not quite forty-eight hours after birth, the nurse noticed that his urine had tinged his napkin a little red, and in the middle of the night, a passage of urine looked decidedly red and bloody. On the morning of the 24th early, I was called to see what had just passed from his urethra, and it seemed almost pure blood, and there was a large amount of it. These passages, perfectly bloody in appearance, continued all day on the 24th and 25th, at intervals of three or four hours, but on the morning of the 26th, the red color began to fade out, and by the evening of the same day, the urine had returned to the natural color, and the child seemed all right again. Sometimes there were small clots passed, and the color during the height of the attack was always of a florid red.

In hæmaturia, it is always difficult to locate the seat of the hemorrhage, and particularly so, in cases of this sort, as it is almost impossible to get urine enough to subject it to any careful analysis. When the hemorrhage is from the ultimate structure of the kidney, we can generally find blood-casts of the uriniferous tubes; and when from the pelvis of the kidney, we can find scales of the renal epithelium, and the blood has not the marked redness which it has when the hemorrhage occurs farther down the passages of the urinary canal. I think it probable, from the very marked redness of the urine in this case, and the frequent occurrence of blood clots in what was passed, that the hemorrhage was mostly from the bladder itself.

The treatment was simply this: With a view of diverting the blood from the central organs to the surface of the body, I had the child put into a bath of water as hot as it could well bear, and afterwards applied mustard poultices to the back, till the skin was well reddened. Internally, I gave $\frac{1}{3}$ gr. of gallic acid in a little syrup and water, every three hours.

I have brought this case to the notice of the Society, because I had never seen one like it before, and because it seemed to me rather an anomalous one. I find nothing of the kind reported in the text-books or journals, nor can I hear of any in the experience of those of my medical colleagues to whom I have mentioned it, with one exception only; my friend, Dr. Ross, has seen one case of the kind, which ultimately recovered under a somewhat similar treatment.

### II. DYSPNŒA; BRONCHIAL SPASM, SIMULATING CROUP.

I was called on the 5th ult. to see a little patient of mine, a boy, five years old, who was said to have a very severe and dangerous choking. I was detained some time, and when I arrived at the house, I found that he had been visited by a neighboring physician, who had pronounced the most unfavorable opinion of him, and had gone away without waiting to see me.

I found the patient in a most critical condition. He was black in the face, throwing his arms wildly about, and making the most frantic efforts to breathe, without being able to get any air into the lungs, and his death seemed very near. As well as I could get at the history and present condition of the case, it seemed certain that the child was suffering from croup, and unless air was admitted into the lungs in some way, he must die very soon. As I had no instrument with me, and desired some assistance and counsel in the matter, I sent for one of the surgeons in the neighborhood. But he could not be found, and my friend, Dr. Ross, was brought in his stead. While the messenger was gone, however, the trouble in breathing seemed a little less, and the color of the lips lost a little of its dusky hue, but they did not become quite red. I noticed, too, that when the boy spoke, which he did now for the first time, not having the power and opportunity before, in his struggle for breath, that his voice was perfectly clear. This threw a new light on the case, and made apparent that there was a spasmodic element in it, and that the larynx itself was not physically and permanently obstructed. There was not, however, any general relaxation or breaking up of the spasm, and when Dr. Ross arrived, the boy seemed about as badly off as ever. It was the opinion of this gentleman, as it had been my own, on first seeing the patient, that suffocation was imminent, and that the child was dying from croup. This was also the opinion of Dr. Baxter, who saw the child soon after, and would have been the opinion of almost any medical man, I think, on first seeing the case. By watching carefully, however, and by the use of anti-spasmodic remedies, we had the pleasure of seeing the child return in a few hours to health again, from a situation which did not seem many minutes removed from death.

The treatment was sedative and anti-spasmodic. In the first place, he was put into a warm bath, then warm fomentations were applied to the chest and leeches to the throat. He took syrup of ipecac. and hive syrup in large doses, without the effect of producing vomiting. Afterwards, he took assafœtida by injection and by the mouth, and this seemed to produce a marked effect on the spasm. He continued to take it all night in alternation with small doses of bromide of potassium, and inhaled chloroform during the most severe periods of the paroxysm. He gradually improved during the night, and was quite well on the 6th, the next morning after his attack.

I have brought up this case to show an interesting point in diagnosis, and to show what possibility there is that a case of this sort might be tracheotomized and claimed as a successful case of that operation in a dangerous and otherwise fatal case of croup. This boy was a very hearty and robust subject, and would undoubtedly have recovered from any operation of the kind. He had had a slight bronchitis for about twenty-four hours previous to this attack, but so slight, that little attention was paid to it. The attack was very sudden and very violent. It may, perhaps, occur to some of you, that it resembled laryngismus stridulus more than anything else, but there was an absence of the crowing inspirations, convulsive movements and other symptoms of that disease. As I said before, it was, at first sight, just like a case of croup, and it was the clearness of the voice which indicated that the trouble was not in the laryngeal structures, but rather in the bronchial tubes, extending, probably, to their minute ramifications. Acting on that supposition, the case was brought to a favorable termination, without resorting to the disagreeable and painful operation of tracheotomy.

